# The Role of Stress Granules in Cardiovascular Diseases

**DOI:** 10.31083/RCM47537

**Published:** 2026-05-18

**Authors:** Xiaoyu Li, Zhongjian Zhang, Luxun Tang, Shuang Li

**Affiliations:** ^1^Department of Cardiology, The General Hospital of Western Theater Command, 610083 Chengdu, Sichuan, China

**Keywords:** stress granules, RNA metabolism, endoplasmic reticulum, mitochondria, Golgi apparatus, lysosome, cardiovascular diseases

## Abstract

Recent research has highlighted the pivotal role of RNA metabolism-related stress responses in the pathophysiology of cardiovascular diseases, particularly atherosclerosis, stroke, atrial fibrillation (AF), and heart failure (HF). Stress granules (SGs) are dynamic, membraneless organelles that arise during RNA metabolism via liquid-liquid phase separation (LLPS), in which mRNA associates with RNA-binding proteins (RBPs). SGs form following translation arrest in response to various external stimuli, resulting in cytoplasmic accumulation of mRNA and RBPs, which subsequently aggregate into membraneless messenger ribonucleoprotein (mRNP) granules, including Cajal bodies, SGs, P bodies, RNA transport granules, and germinal bodies. This review focuses specifically on SGs. SG formation is typically a transient and protective cellular response to stress; however, the dysregulation or persistence of SG formation has been implicated in a range of diseases, including cardiovascular conditions, neurodegenerative disorders, cancers, immune responses, and viral infections. Thus, this review examines the physiology and pathology of SGs, detailing the associated formation, composition, regulation, and function, with a particular focus on the involvement of SGs in cardiovascular diseases (CVDs) and potential therapeutic strategies targeting SGs. Moreover, this review outlines the complete life cycle of SGs and the associated implications in CVD. SGs originate near the endoplasmic reticulum (ER) and mitigate apoptosis by curbing mitochondrial production of reactive oxygen species. SGs can also disrupt the trafficking of specific cargo from the ER to the Golgi apparatus. Furthermore, SGs can repair damaged lysosomes and eventually undergo self-clearance via the autophagy-lysosome pathway. This model provides new perspectives for researchers in cardiovascular medicine, physicians, and translational medical researchers, and may advance our understanding of SG-related pathophysiology and facilitate the identification of novel therapeutic targets for CVDs.

## 1. Introduction

Cardiovascular diseases (CVD) are one of the most significant causes of mortality worldwide. The traditional risk factors for cardiovascular diseases (gender, smoking, diabetes, systolic blood pressure [SBP], non-high-density lipoprotein [HDL] cholesterol, and body mass index [BMI]) are associated with a high incidence of cardiovascular diseases. There are significant differences in the incidence and prevalence of CVD across various geographical locations. Non-traditional risk factors include unhealthy lifestyles, new biomarkers, certain adverse disease states, environmental factors, inflammation, and oxidative stress. The metabolism of RNA has attracted significant attention as a non-traditional factor in the cardiovascular system, and it plays a crucial role in various physiological and pathological processes [1, 2]. These include modulating inflammatory responses, fostering angiogenesis, influencing cell proliferation and migration, and driving pathological processes such as fibrosis.

The precise regulation of macromolecular localization and utilization is fundamental to cellular biology. Typically, molecules are compartmentalized into organelles encased by lipid membranes, as seen in the endoplasmic reticulum (ER), mitochondria, lysosomes, Golgi apparatus, and other cellular entities [3, 4, 5]. However, RNA is seldom enveloped by lipid membranes; instead, RNA localization is predominantly governed by its binding to RNA-binding proteins (RBPs) and these RBPs can aggregate through liquid-liquid phase separation (LLPS), giving rise to membrane-less organelles known as RNA granules [6, 7, 8, 9]. Among the diverse RNA granules, stress granules (SGs) appear to be linked to CVD. Mutations exist in several genes encoding RBPs involved in the SG response. In animal models of CVD, interventions that curb SG accumulation have demonstrated the potential to alter disease progression. This article summarizes recent research findings and reviews the role of SGs as potential therapeutic targets in CVD, due to their involvement in intracellular generation and degradation pathways.

The following discussion centers on key cardiovascular and related conditions: atherosclerosis, stroke, sepsis, atrial fibrillation (AF), heart failure (HF), pulmonary arterial hypertension (PAH), and myocardial ischemia-reperfusion (MIR). Atherosclerosis manifests as lipid accumulation in arteries, coupled with smooth muscle cell proliferation and atherosclerotic calcification, gradually evolving into atherosclerotic plaques [10, 11]. SGs emerge within arterial vascular smooth muscle cells (VSMCs) as well as the macrophages of these plaques, and contribute to disease progression. Treatment strategies focus on plaque removal and clearance of SGs. Stroke arises from inadequate cerebral blood and oxygen supply, mainly due to intracranial artery stenosis or middle cerebral artery occlusion (MCAO). It is mainly associated with the aggregation of ribonucleoproteins such as hnRNPA0, hnRNPA1, DNA-binding protein-43 (TDP-43), and fused in sarcoma (FUS) in neurons [12]. Sepsis is an acute and life-threatening condition marked by organ dysfunction due to a dysregulated host response to infection, featured by excessive inflammation, catabolism, metabolic disturbances, and immunosuppression [13]. AF is a persistent arrhythmia with irregular and abnormal heartbeats. Its development is associated with oxidative stress, inflammation, and fibrosis. Hypoxia-inducible factor (HIF)-1α plays a pivotal role in these processes and can induce SG formation, offering protection against AF [14, 15]. HF is primarily driven by cardiac remodeling, involving myocardial hypertrophy and fibrosis, and is associated with abnormal cytoplasmic accumulation of RNP granules, particularly RBM20 RNP granules with liquid-like properties [16]. HF may be linked to aberrant SG accumulation. PAH is characterized by increased pulmonary vascular resistance and pathological remodeling of the pulmonary artery, leading to right ventricular hypertrophy. Elevated expression of long non-coding RNA LINC00599 in the pulmonary artery medial layer may regulate SG formation. MIR is characterized by myocardial dysfunction, structural damage, and electrical activity disorders after the restoration of coronary artery blood flow in ischemic heart disease [17, 18].

## 2. Membrane-Less Organelle RNA Granules

**Fig. 1.**
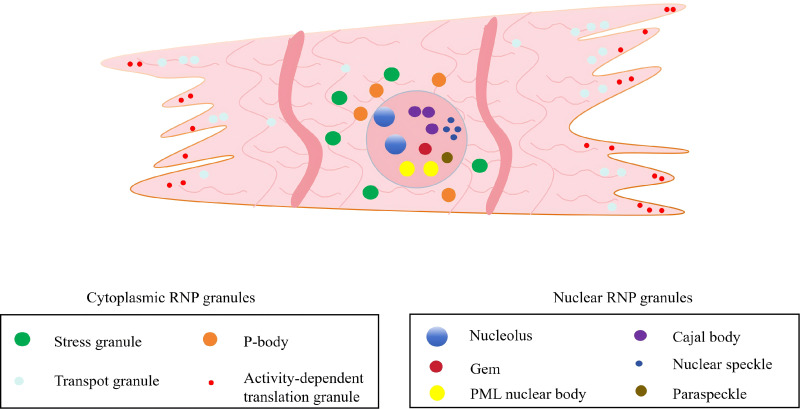
**The distribution of ribonucleoprotein (RNP) granules within the CMs**. Membrane-less organelles are ubiquitous in CMs. In the cytoplasm of CMs, RNA granules include SGs P-bodies, transport granules, and activity-dependent translation granules. In the nucleus of CMs, the main RNA granules are the nucleolus, Cajal bodies, Gems, nuclear speckles, PML nuclear bodies, and paraspeckles. A list of RBPs that shuttle between the nucleus and cytoplasm, as well as those mainly located in the cytoplasm. These membrane-less organelles exist in CMs or in CVD. CMs, cardiomyocytes; SGs, stress granules; PML, promyelocytic leukemia; RBPs, RNA-binding proteins; CVD, cardiovascular diseases. The figure was created by biorender (https://www.biorender.com).

In mammalian eukaryotic cells, the interior is compartmentalized into distinct regions known as organelles [19, 20]. These organelles are delineated and encapsulated by membranes composed of phospholipid bilayers, which not only establish their boundaries but also isolate their internal environment from the cytoplasm. Each organelle harbors a unique set of enzymes and specific molecules, enabling precise temporal regulation of diverse biochemical processes [21, 22]. Interactions among proteins in different regions enable organelle identification and promote efficient cellular function. These organelle membranes are integral not only to the organelles themselves but also facilitate communication within the cell, such as the transfer of life-sustaining substances between organelles and their surroundings, as well as among different organelles, thereby integrating various cellular activities [23, 24, 25]. Typical examples of lipid-encapsulated organelles include the nucleus, mitochondria, ER, and the Golgi apparatus. Another stable cellular compartment consists of membrane-less organelles, which are formed through the association of RBPs through LLPS in RNA [26]. These membrane-less structures are collectively referred to as RNA granules [27]. RNA granules are present in the cytoplasm and the nucleus. The primary types of cytoplasmic RNA granules include SGs, P-bodies, transport granules, and activity-dependent translation granules [27, 28] (Fig. 1). In the cardiomyocytes (CMs) nucleus, the main RNA granules are the nucleolus, Cajal bodies, Gems, nuclear speckles, promyelocytic leukemia (PML), nuclear bodies, and paraspeckles (Fig. 1). Specific molecules are concentrated within these liquid compartments, which coexist with the surrounding liquid environment. Protein LLPS is driven by inherent disordered regions (IDRs) of the components as well as modular interaction domains [29]. Membrane-less organelles appear to support specific biochemical processes and play crucial roles in maintaining cellular homeostasis and development [30]. SGs, in particular, represent a specialized protective mechanism employed by cells in response to environmental stresses such as arsenite exposure, hypoxia, and heat shock [31, 32]. During these stress conditions, cellular translation and apoptosis are inhibited, causing DNA damage and the accumulation of misfolded proteins, which aggregate in the cytoplasm and form SGs along with mRNA, translation initiation factors, various RBPs, and non-binding proteins [33].

## 3. The Dynamics of SGs: Assembly, Disassembly, Fusion, Coupled With Fission

**Fig. 2.**
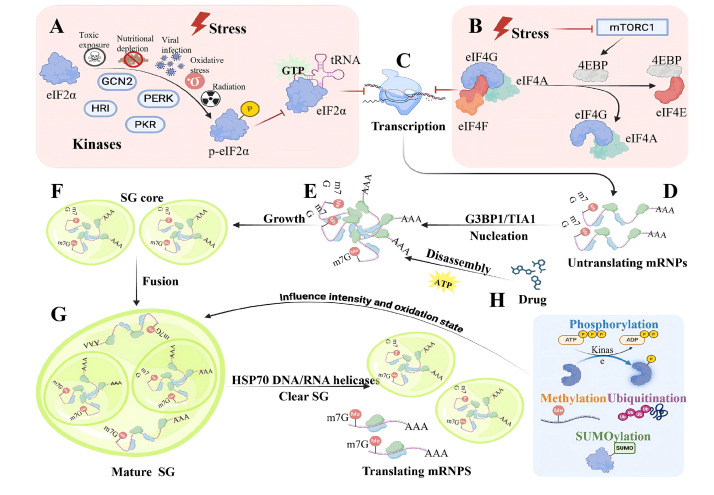
**The Dynamics of SGs: assembly, disassembly, fusion, coupled with fission**. The formation of SG is triggered by the inhibition at the beginning of translation. This process is regulated by two major stress response signaling pathways: (A) eIF2α phosphorylation. Under stress conditions, specialized kinases such as *pERK*, *GCN2*, *PKR*, and *HRI* phosphorylate eIF2α, thereby preventing the formation of the eIF2 trimer complex. (B) Under pressure conditions, mTORC1 is inactivated, reducing the phosphorylation level of 4EBP. This enables 4EBP to bind to eIF4E, thereby disrupting the eIF4F complex. (C) Both of these pathways hinder the initiation of translation. (D,E) The deposited mRNP complexes attract nuclearators such as GTPase-activating protein SH3 domain-binding protein (G3BP)1/2 and T-cell intracellular antigen 1 (TIA1), forming the initial core part of the SG structure. (F) These SG seeds fuse and mature into higher-order structures by attracting shell components. Colored ellipses represent RNA-binding proteins, wavy lines represent RNA, and dashed lines represent weak interactions between mRNPs in the SG shell. (G) The disintegration stage of the SGs. After stress is relieved, the SGs begin to disintegrate by first dissolving the shell and then dispersing the core. This process allows mRNPs to re-enter a recovering translation mRNP pool. Some persistent-SGs are encapsulated in autophagosomes and eventually degraded through the autophagy pathway. The disintegration of SG regulated by HSP70, DNA/RNA helicases, and ATP. (H) The reduction of ATP levels by the drug will prevent the movement, fusion and division of lysosomes. GCN2, general control nonderepressible 2; HRI, heme-regulated inhibitor; PERK, PKR-like endoplasmic reticulum kinase; PKR, protein kinase R; GTP, guanosine triphosphate; 4EBP, eukaryotic translation initiation factor 4E-binding protein; TIA1, T-cell intracellular antigen 1; HSP70, heat shock protein 70; ATP, adenosine triphosphate; ADP, adenosine diphosphate. The figure was created by biorender (https://www.biorender.com).

LLPS is highly responsive to environmental changes that influence multivalent interactions. Variables such as composition, concentration, temperature, pH, salt concentration, and post-translational modifications (PTMs) of proteins, including phosphorylation, methylation, ubiquitination, as well as sumoylation, can change the strength and valence of these interactions [34, 35, 36]. Over time, the highly dynamic components of the liquid condensate transform into a thermodynamically more stable environment [37]. Consequently, unlike membrane-bound organelles, the assembly of condensates is highly dynamic and reversible [38]. The formation of SGs is also dynamic and reversible, enabling rapid responses to cellular dysfunction. In eukaryotic cells, various cellular stresses, such as arsenic, hypoxia, and heat shock, can inhibit mRNA translation initiation and disassemble polysomes, resulting in the cytoplasmic accumulation of untranslated, 80S ribosome-free mRNA [20, 39]. These mRNAs bind to RBPs and aggregate into micron-sized, non-membrane-bound organelles known as SGs. SGs represent a specialized protective mechanism that cells employ in response to environmental stresses, including arsenite exposure, hypoxia, and heat shock. Under these conditions, cellular translation and apoptosis are inhibited, resulting in DNA damage and the accumulation of misfolded proteins. These substances, along with mRNA, translation initiation factors, and various RBPs, coupled with non-binding proteins, aggregate in the cytoplasm to form SGs (Fig. 2).

The formation of SG is closely related to translation inhibition. When translation is inhibited, the polyribosome will deplete its mRNA, and the “exposed” mRNA will assemble with the SG nucleating protein to form LLPS. Under non-stress conditions, cells undergo phosphorylation of eukaryotic translation initiation factor 2α (eIF2α) a process mediated by four kinases: general control nonderepressible 2 (GCN2), protein kinase R (PKR), heme-regulated inhibitor (HRI), and PKR-like endoplasmic reticulum kinase (PERK), which impedes the formation of a functional ternary Eukaryotic translation initiation factor 2-GTP-initiator tRNA complex (eIF2-GTP-tRNA) complex and leads to the accumulation of untranslatable initiation complexes that subsequently lead to the formation of SGs [40, 41] (Fig. 2A). GCN2 monitors homeostatic amino acid/charged tRNA levels by binding to deacylated tRNA [42]. PKR is primarily activated by binding to double-stranded RNA and plays a major role in the cellular antiviral response. PKR can be activated by the protein activator PACT under conditions such as serum starvation, peroxide exposure, and arsenite treatment [43, 44]. HRI responds to heme deficiency, oxidative stress, and heat shock [45]. PERK is an ER transmembrane kinase with an ER cavity unfolded protein-sensing domain and a cytoplasmic eIF2α kinase domain [46, 47]. PERK activation occurs in response to the accumulation of unfolded proteins in the ER, a scenario commonly observed during viral infections, which also induces SG formation [48]. In response to viral infections, organisms have evolved to produce IFN antiviral signals that interfere with the assembly of SG and weaken viral replication. Both of these processes rely on PERK activity, indicating that SGs depend on PERK to play a crucial role in antiviral defense [49].

Another mechanism that operates under pressure conditions, cap-dependent translation, is initiated by the assembly of the eukaryotic translation initiation factor 4F (eIF4F) complex (eIF4E, eIF4G, eIF4B, and eIF4A) on the 5^′^ 7-methylguanosine cap (5^′^ cap) of the mRNA. A key regulatory event in the assembly of the eIF4F complex is the phosphorylation of the eukaryotic translation initiation factor 4E-binding protein (eukaryotic translation initiation factor 4E-binding protein [EIF4EBP], also known as eukaryotic translation initiation factor 4E-binding protein [4EBP]), which competes with eIF4G for binding to eIF4E and prevents the assembly of the eIF4F complex. Under stress conditions, mTORC1 is inhibited, and the phosphorylation of eIF4EBP can prevent the binding of eIF4EBP and eIF4E and promote the formation of the eIF4F complex [50, 51] (Fig. 2B). Untranslated mRNP complexes accumulate in the cytoplasm (Fig. 2C). These accumulated mRNP complexes subsequently recruit specific RNA-binding proteins, namely SG initiation factors, thereby forming the initial structure of SG. These SG initiation structures then fuse and grow into mature structures by recruiting more protein components rich in IDRs (Fig. 2D–F).

SGs are dynamic entities characterized by liquid-like properties, rapid component exchange rates, decomposition into translated mRNP, and clearance via autophagy [52, 53]. The dynamic behavior of SGs is driven by adenosine triphosphate (ATP)-dependent remodeling complexes. Acute pharmacologic depletion of ATP abolishes SG movement, fusion, and fission [54] (Fig. 2H). In addition, protein exchange within SGs is ATP-dependent, as evidenced by the inability to restore Ras-GTPase-activating protein SH3 domain-binding protein (G3BP) after photobleaching when ATP levels are depleted [55]. Stress granular proteins and mRNA can form stable interactions that are disrupted by ATPase. During stress, when the concentration of granule components, such as untranslated RNA, is high, ATPase transiently disrupts these interactions, facilitating rapid component exchange. Upon recovery, this disruption leads to SG disassembly, with autophagy potentially clearing residual material. ATPases involved in SG dynamics may include complexes that directly influence interactions within SGs, such as protein chaperones and RNA helicases [56]. SG depolymerization is also linked to molecular chaperons like heat shock protein 70 (Hsp70), which may boost SG disintegration by inhibiting the accumulation of misfolded proteins within SGs [57] (Fig. 2G). Helicases also contribute to SG disassembly; for example, RNA/DNA helicases use the energy from ATP hydrolysis to displace proteins or nucleic acids bound to nucleic acids, thereby regulating the breakdown of SGs [58, 59] (Fig. 2G).

## 4. The Connection Between SGs and Other Organelles

### 4.1 Endoplasmic Reticulum (ER)

**Fig. 3.**
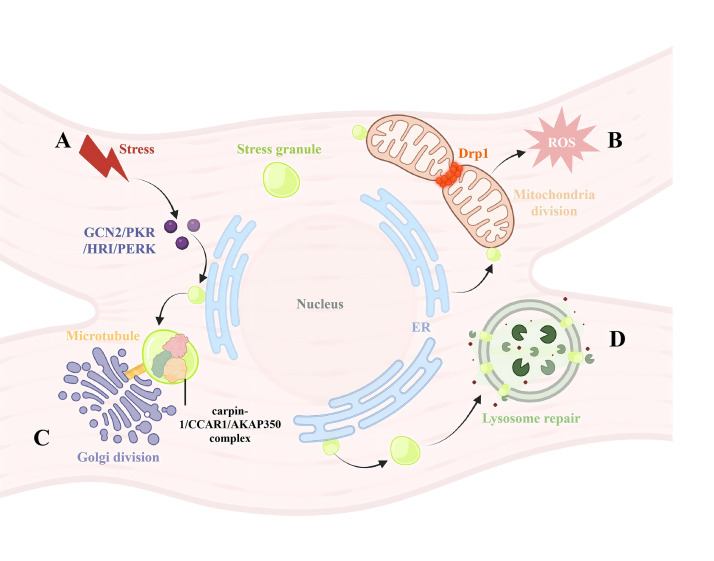
**The connection between SGs and other organelles**. (A) Stress factors will cause phosphorylation of eIF2α in the endoplasmic reticulum, thereby blocking overall protein synthesis and enhancing the protein folding and degradation capabilities of the endoplasmic reticulum. Eventually, this leads to the accumulation and formation of SGs, which are the sites where SGs are produced. (B) During the process of SG activity, mitochondria provide energy. Stress causes the fragmentation of mitochondria, and this process is highly dependent on Drp1, which is closely related to the increase in reactive oxygen species release and calcium overload. (C) After treatment with sodium arsenite or heat shock, AKAP350 binds to RNA-binding protein caprin-1 and CCAR1, forming a complex. These complexes are localized on the smooth endoplasmic reticulum and co-localize with G3BP, thereby causing the division of the Golgi apparatus stained with GM130. This process depends on a complete microtubule network. (D) SGs act as a “plug” to prevent the leakage of substances in the lysosomes, and promote lysosome repair through mechanisms dependent on ESCRT and independent mechanisms. Drp1, dynamin-related protein 1; ROS, reactive oxygen species; AKAP350, A-kinase anchoring protein 350; GM130, Golgi matrix protein of 130 kDa; CCAR1, cell cycle and apoptosis regulator 1. The figure was created by biorender (https://www.biorender.com).

The ER serves as the central organelle in the eukaryotic secretory pathway, primarily tasked with protein folding, biosynthesis, translocation, and PTMs such as glycosylation, disulfide bond formation, and chaperon-mediated protein folding. Stressor triggers eIF2α phosphorylation in the ER, which blocks overall protein synthesis and subsequently enhances the ER’s capacity for protein folding and degradation, ultimately leading to the accumulation and formation of SGs [60, 61] (Fig. 3A). During ischemia/reperfusion (I/R), hypoxia, and increased free radical exposure in brain cells disrupt ER homeostasis and diminish its protein-folding capacity, causing unfolded proteins to accumulate and aggregate within the ER [62]. In mammalian cells, under various endoplasmic reticulum stress conditions, inositol-requiring enzyme 1 (IRE1) is activated, and it will splice *XBP1* mRNA, initiating the endoplasmic reticulum response pathway, resulting in a phase separation of the endoplasmic reticulum membrane. IRE1α can dynamically form co-localization with SG through its disordered region in the cytoplasmic connection structure, forming IRE1α aggregates [63]. To restore cellular homeostasis after ER stress, a series of reactions known as the unfolded protein response (UPR) is initiated [64]. The primary role of UPR is to temporarily halt translation of ER-targeted mRNAs. UPR signal transduction can be categorized into three pathways: Inositol-requiring enzyme 1α (IRE1α), PERK, and activated transcription factor 6 (ATF6) [30]. During heat shock, K63-linked ubiquitination of G3BP1 facilitates its interaction with the ER-associated protein fas-associated factor 2 (FAF2), which recruits segregase valosin-containing protein (VCP)/p97 to extract G3BP1 from SGs, causing their disassembly. These pathways collectively inhibit mRNA translation under ER stress, preventing further protein synthesis, and thereby reducing the protein-folding burden to alleviate stress [65].

### 4.2 Mitochondria

Mitochondria serve as the primary energy source for cardiac smooth muscle cells and play a pivotal role in regulating cardiovascular smooth muscle by influencing reactive oxygen species (ROS) formation, mitochondrial DNA (mtDNA), and calcium-mediated metabolism [66, 67].

The formation of SGs acts as an antagonistic mechanism against apoptosis. SGs inhibit apoptosis by reducing ROS generation, functioning as key redox regulators that determine cell survival under stress conditions [68]. Proteins associated with SGs, such as G3BP1 and USP10, control their antioxidant activity [69]. Common signal transduction pathways involving ROS include phosphoinositide 3-kinase (PI3K), mitogen-activated protein kinase (MAPK), coupled with protein kinase C (PKC) [70]. Abnormal activation of the oncogenic PI3K/mTOR pathway in ovarian cancer cells is crucial for tumor resistance. Treatment with dual PI3K/mTOR inhibitor PKI-402 induces SG formation in A2780 cells and intercepts activating transcription factor 5 (ATF5), preventing its entry into the nucleus to regulate the mitochondrial unfolded protein response [71] (Fig. 3B). TM4SF1 antisense RNA 1 (TM4SF1-AS1) promotes SG formation in cells, inhibits apoptosis, and facilitates tumorigenesis by sequestering receptor for activated c kinase 1 (RACK1), an activator of the stress-responsive MAPK pathway, within SGs [72]. Histone deacetylase (HDAC) is an important regulator of ROS signaling and cellular responses; the HDAC inhibitor trichostatin A prevents H_2_O_2_-induced SG formation [73].

mtDNA and nuclear DNA can translocate to the cytoplasm, where they bind to “DNA sensors” and initiate signaling cascades. G4DNA promotes SG formation by interacting with RNA-binding proteins, which accumulate in the cytoplasm. TDP-43 is a major component of inclusion bodies in frontotemporal lobe degeneration (FTLD-U) as well as amyotrophic lateral sclerosis (ALS) [12]. While TDP-43 is primarily located in the nucleus, it undergoes abnormal processing in affected areas of the central nervous system in ALS and FTLD-U, forming cytoplasmic inclusion bodies [74]. Evidence suggests that TDP-43 regulates mRNA metabolism by interacting with mRBPs associated with RNA granules.

During nutrient deprivation, mammalian cells will reorganize their metabolic networks, shifting glucose metabolism toward lipid utilization. SGs formation contributes to this metabolic reprogramming by downregulating fatty acid beta-oxidation (FAO) through the regulation of mitochondrial voltage-dependent anion channels (VDACs), thereby introducing fatty acids (FAs) into the mitochondria to maintain energy levels [75].

Mitochondrial SGs are closely associated with the inner mitochondrial membrane and rely on mitochondrial dynamics for their formation and function. Ischemia induces mitochondrial fragmentation, a process heavily dependent on dynamin-related protein 1 (Drp1), which is associated with increased ROS release and calcium overload [76, 77, 78]. Bβ2 acts as a neuron-specific activator of Drp1 *in vivo*. Loss of Bβ2 preserves Drp1 phosphorylation at Serine 637 (Ser637), thereby enhancing mitochondrial respiration, maintaining calcium homeostasis, and reducing superoxide formation, ultimately protecting the brain from stroke injuries [79] (Fig. 3B). Drp1 activation and subsequent mitochondrial fission contribute to ischemic brain damage. Genetic disruption of mitochondrial protein kinase A (PKA) scaffolding by knocking out *AKAP1* exacerbates stroke injury in mice, since the extramitochondrial A-kinase anchor protein 1 (AKAP1)/PKA complex normally inhibits Drp1-dependent mitochondrial fission to protect neurons from ischemic stroke by maintaining respiratory chain activity, inhibiting superoxide formation, along with delaying Ca [79].

### 4.3 The Golgi Apparatus

Under stress conditions, components of the Golgi apparatus or molecules involved in Golgi cargo transport can be recruited into SGs, leading to Golgi fragmentation. This process depends on an intact microtubule network, since microtubule disruption inhibits the formation of cytoplasmic ribonucleoprotein SGs.

A-kinase anchoring protein 350 (AKAP350A) (also known as AKAP450 or CG-NAP) is a multifunctional scaffold protein localized to the Golgi apparatus as well as the centrosome. Upon treatment with sodium arsenite or heat shock, AKAP350 binds to the RNA-binding proteins caprin-1 and cell cycle and apoptosis regulator 1 (CCAR1), forming complexes that localize to SGs and colocalize with G3BP, resulting in the fragmentation of Golgi matrix protein of 130 kDa (GM130)-stained Golgi structures, a process that depends on an intact microtubule network [80] (Fig. 3C).

*PARP12*, a single ADP-ribosyltransferase (mART) localized to the Golgi complex, relocates to SGs under sodium arsenite or heat shock conditions. During stress, nuclear PARP1 is activated, and endogenous Poly (ADP-ribose) polymerase 12 (PARP12) translocates from the Golgi to SGs, where it localizes through direct binding to PAR. The co-localization of the SG marker G3BP with the TGN marker Golgin-97 decreases, and SG components move along intact microtubules. This process is dynamically regulated, with PARP12 returning to the Golgi complex upon stress removal [81].

Under stress conditions such as sodium arsenite and heat shock, the transport protein particle (TRAPP) complex is recruited into SGs, controlling their maturation. The sequestration of coat protein complex II (COPII)/TRAPP complexes in SGs prevents cargo transport from the ER to the Golgi apparatus. The removal of the stressor releases these complexes, restoring transport. The Parkinson’s disease-related protein GRB10-interacting GYF protein 2 (GIGYF2), which is associated with COPII vesicular proteins and localizes to the ER and the Golgi in resting cells, is driven into SGs following arsenite treatment, highlighting its role in vesicular transport between these organelles [82].

### 4.4 Lysosomes

Lysosomes, membrane-bound organelles crucial for degradation and signal transduction, have recently been found to interact dynamically with SGs. Following endolysosomal damage induced by the L-leucyl-l-leucine methyl ester (LLOMe), SGs selectively form and accumulate biomolecular condensates near the damaged lysosomal membrane. SGs act as a “plug”, preventing lysosomal contents from leaking out and facilitating lysosomal repair through both ESCRT-dependent and independent mechanisms (Fig. 3D) [83].

Lysosomes are commonly considered the primary platform for tuberous sclerosis complex (TSC) complex-mediated inhibition of mechanistic target of rapamycin complex 1 (mTORC1). Lysosomal injury triggers SG formation and inactivation via the TSC-MTOR axis, with damaged lysosome signals transduced to SGs through the lysosome-resident *EIF2AK2/PKR* pool [84]. Phosphorylation of EIF2A inhibits capped mRNA translation, leading to SG formation and an ATF4-driven comprehensive stress response to lysosomal damage. Lysosomal injury inactivates mTOR, while damaged lysosomes undergo GABA type A receptor-associated protein (GABARAP)-mediated Atg8ylation, which directly recruits the SG proteins nuclear FMRP-interacting protein 2 (NUFIP2) and G3BP1 to the lysosomal surface, further contributing to mTOR inactivation [85]. G3BP1 and G3BP2, core SG components, reside on the cytoplasmic surface of lysosomes, where the TSC protein complex is anchored through activation of the mTORC1 metabolic regulatory factor. High-density lipoprotein-binding protein (HDLBP), identified in two omics analyses of SGs and recently confirmed to localize to SGs in mammalian cells, mediates SG recruitment of TSC2, as knockdown of HDLBP reduces TSC2 localization to SGs [86].

The relationship between lysosomes and SGs also sheds light on autophagy-mediated SG clearance and assembly. mTORC1, a key inhibitor of autophagy, suppresses autophagosome initiation by inhibiting autophagy-activating UNC-51-like kinase 1 (ULK1) and Autophagy related protein 13 (ATG13), as well as the major transcription factors transcription factor EB (TFEB) and transcription factor E3 (TFE3) in the autophagolysosomal pathway [87, 88]. *NEDD4*, a major E3 ligase involved in protein quality control after heat stress, binds to the Hsp40-Ydj1 complex, which colocalizes with SGs following heat stress and is essential for SG clearance [89].

## 5. Research and Progress of SGs in CVD and Other Diseases

### 5.1 Atherosclerosis

Atherosclerosis is a common, chronic CVD with high morbidity and mortality. It is characterized by arterial lipid accumulation, smooth muscle cell and fibrous matrix proliferation, vessel wall thickening, elastin degradation, stromal “Browning”, endothelial dysfunction, medial and atherosclerotic calcification, and progressive plaque formation [90]. G3BP1 serves as a major determinant of the severity of CVD, as evidenced by angiographic studies. *In vivo* studies using LDLR-/- mice, an atherosclerotic disease model, revealed that SGs form within atherosclerotic plaques in aortic VSMCs as well as macrophages as the disease progresses, with SG formation positively correlating with the severity of the disease [91]. *In vitro* experiments demonstrated that exposing VSMCs and bone marrow-derived macrophages to various atherosclerotic plaque stimuli, such as oxidized low-density lipoprotein, mitochondrial stress, and oxidative stress mediators, leads to increased eIF2 phosphorylation and rapid induction of SG [91]. SG formation appears to be a common response to inflammation caused by vascular injury and represents a transcriptional adaptation of the vasculature to local inflammation, which enables VSMCs and other cell types involved in vascular inflammation to isolate mRNA transcripts, halting their translation as well as forming RNP granules such as SGs [11] (Table 1, Ref. [10, 11, 17, 18, 92, 93, 94, 95, 96, 97, 98, 99, 100, 101, 102]).

**Table 1.** **Summary of SG defects in various CVD and other diseases**.

Disease types	Disease proteins	SG localization (expression/sample/stressor/SG marker)	SG defects associated with disease (defects)	Evidence in cardiovascular system: yes/no	References
Atherosclerosis	/	Perinuclear or in cytoplasmic lamellae/Primary human vascular cell/oxLDL, clotrimazole/PABP, HuR, and FXR1	Pro-atherosclerotic stimulate stress granule formation in plaque macrophages and VSMC.	yes	[11]
Atherosclerosis	G3BP2	Endogenous/Endothelial cells/ox-LDL/G3BP2	Reduced SG formation.	yes	[92]
The acute ischemic stroke (AIS)	METTL3/miR-335	Endogenous/cortex, PC12 cells, and primary cortical neurons/OGDR/(oxygen-glucose deprivation/recovery)/G3BP1, TIA1	METTL3/miR-335/Erf1 axis promotes SG Formation might reduce AIS injury in the early stage of the disease.	no	[95]
The acute ischemic stroke (AIS)	ROCK2	Endogenous/MCAO rats and PC12 cells/serum-freestimulation/TIA1, G3BP1	MiR-335 promotes SG formation by targeting the mRNA of ROCK2.	no	[94]
The acute ischemic stroke (AIS)	RBM3	Endogenous/primary cortical neurons and PC12 cells/OGDR/TIA1, G3BP1	RBM3 promotes the formation of lysosomes by binding to G3BP1, thereby enhancing the anti-apoptotic ability of PC12 cells under oxygen-glucose deprivation.	no	[100]
Ischemic stroke	TDP-43	Endogenous/mice brain tissue/middle cerebral artery occlusion (tMCAO)/G3BP1, TDP-43	TDP-43 mislocalized by ischemia may be incorporated into SGs that form around G3BP1 by binding to HDAC6, and ultimately becoming degraded.	no	[93]
Sepsis	/	Overexpression/Newborn wild-type mice cardiomyocytes/LPS/G3BP1 and TIA-1	SG exert a protective effect against CM dysfunction in sepsis.	no	[96]
Sepsis	YB-1	Endogenous/HEK293T/sodium arsenite, H_2_O_2_/PABP1	YB-1 contributes to the formation of SGs (SGs).	no	[97]
Myocardial infarction (MI)	HSF1	Endogenous/mice cardiomyocytes/Metformin/FMRP, G3BP, FXR1, and DDX3	Metformin stimulated cardiomyocytes to produce SGs through HSF1.	yes	[101]
AF	/	Overexpression/HL-1 cells, cardiomyocytes and cardiac fibroblasts/Paced/G3BP1, PABP-1	G3BP1 overexpression promotes SG formation against oxidative stress, calcium overload and atrial fibrosis in AF.	yes	[10]
PAH	LINC00599	Endogenous/mouse lung tissues and pulmonary arterial smooth muscle cells (PASMCs)/hypoxic conditions/G3BP1	LINC00599 regulates LLPS through m6A modification, promoting the proliferation of PASMCs.	yes	[18]
PAH	/	Endogenous/human PAH tissues, pulmonary artery smooth muscle cells in rats, Sugen, Hypoxia, arsenite/Caprin1, G3BP1	SG formation contributes to abnormal vascular phenotypes.	yes	[17]
PAH	CircSSR1	Endogenous/pulmonary artery smooth muscle cells/hypoxia/G3BP1	As the upstream of circSSR1, G3BP1 promotes the degradation of circSSR1 under hypoxic conditions.	yes	[102]
Myocardial ischemia-reperfusion (MIR)	Sephin1	Endogenous/H9c2 cells/hypoxia-reoxygenation/G3BP1	Sephin1 enhances ISR in H9c2 cells after H/R *in vitro*.	yes	[98]
MIR	ALKBH5	Endogenous/H9c2 rat cardiomyocyte cell/high glucose hypoxia/reoxygenation/G3BP1	ALKBH5 might inhibit the apoptosis of cardiomyocytes by promoting the expression of SGs through the cGAS-STING pathway.	yes	[99]

VSMC, vascular smooth muscle cells; PABP, poly(A)-binding protein; HuR, human antigen R; FXR1, fragile X-related protein 1; G3BP2, Ras-GTPase-activating protein SH3 domain-binding protein; METTL3, methyltransferase-like 3; ROCK2, rho-associated coiled-coil-containing protein kinase-2; OGDR, oxygen-glucose deprivation/reperfusion; TDP-43, DNA-binding protein-43; LLPS, liquid-liquid phase separation; PAH, pulmonary arterial hypertension; RBM 3, RNA-binding motif protein 3; LPS, lipopolysaccharide; HSF1, heat shock factor 1; cGAS-STING, cyclic GMP–AMP synthase-stimulator of interferon genes.

Research has revealed that G3BP1 arginine methylation hinders SG assembly, while the absence of LRP6 in VSMCs promotes Wnt signaling, and G3BP1 interacts with RIG-I, enhancing its own arginine methylation. The G3BP1/RIG-I/MAVS axis plays a pivotal role in osteogenic differentiation, aortic VSMC mineralization, and aortic calcium accumulation. Gpr137b-ps regulates amino acid-Mtorc1-autophagy signaling by inhibiting the interaction between HSC70 and G3BP, providing a potential therapeutic strategy for advanced atherosclerosis [103] (Table 1). The G3BP1 homolog G3BP2, another core SG protein, is highly expressed in inflammatory endothelial cells. Inhibiting G3BP2 in these cells diminishes oxidized low-density lipoprotein (OX-LDL)-induced inflammation and activates the nuclear factor kappaB (NF-κB) signaling pathway. G3BP2 knockdown reduces early atherosclerotic plaque formation in *ApoE*^-⁣/-^ mouse models of atherosclerosis, making it a promising therapeutic target. Immunization against G3BP2 reduces the number of macrophages and levels of pro-inflammatory cytokines. Glutamine supplementation may mitigate atherosclerosis by downregulating O-GlcNAc modification, glycolysis, oxidative stress, and pro-inflammatory pathways [92]. The levels of G3BP2 expression are very high in inflammatory endothelial cells. Knocking down G3BP2 in *ApoE*^-⁣/-^ mice reduces the development of atherosclerosis. SGs are pathogenic in atherosclerosis.

Actin polymerization and cytoskeleton-associated proteins localize to SGs and participate in their dynamic regulation, indicating that the cytoskeleton of VSMCs may play a vital role in controlling SG formation as well as cellular stress responses. Activated by various pathophysiological stressors, including hypoxia, oxidized lipids, coupled with inflammation, and expressed in *LDLR*^-⁣/-^ mice with atherosclerotic plaques, calpain (acute calpain, as referred to in context) markedly ameliorates cardiovascular dysfunction by reducing both acute and chronic inflammation. In addition, cells expressing the calpain inhibitor exhibit impaired ability to extend lamellar pseudopodia, along with abnormalities in filamentous pseudopodia elongation and contraction, indicating that calpain may modulate SG dynamics and have a significant impact on SG formation in VSMCs during initiation and development of atherosclerosis and restenosis [104, 105] (Table 1).

### 5.2 Stroke

The definition of stroke includes ischemic stroke (cerebral infarction) as well as hemorrhagic stroke (encompassing parenchymal, ventricular, and subarachnoid hemorrhage). Ischemic stroke results from inadequate blood and oxygen supply to the brain, primarily due to intracranial artery stenosis and MCAO. Cerebral ischemia results in diverse secondary effects, such as reperfusion injury, ischemic hypoxia, intracellular calcium overload, blood-brain barrier disruption, reactive oxidative stress, apoptosis, inflammation, as well as ion imbalances, culminating in neuronal damage.

The kinetic characteristics of SGs assembly following cerebral ischemia may confer protection against the delayed progression of ischemic brain injury. In a mouse model of cerebral ischemia, cortical aggregation of RBPs, including ribonucleoprotein hnRNPA0, hnRNPA1, hetero-ribonucleoprotein P2 (FUS) TAR, and TDP-43, was observed one hour after reperfusion [93, 106]. These RBPs translocated from the nucleus to the cytoplasm but dissolved within 24 hours. Although existing studies have not directly elucidated the kinetic relationship between ischemic stroke and SGs, it can be inferred that SGs form to protect mRNA during ischemia. Upon the restoration of oxygen supply, these SGs disassemble, and RBPs return to the nucleus, enabling the resumption of translation. However, if stress persists, as in permanent MCAO or hemorrhagic stroke, RBPs remain aggregated, preventing SG disassembly and the resumption of translation, eventually leading to neuronal apoptosis. MCAO, along with serum-free cell models, has simulated diverse pathological characteristics of early stroke, revealing a negative correlation between SG formation and apoptosis levels in MCAO rats [93] (Table 1). miR-335-promoted SG formation inhibited apoptosis by inhibiting rho-associated coiled-coil-containing protein kinase-2 (ROCK2) expression in AIS [94]. These observations suggest that reducing SG formation may promote apoptosis levels as well as exacerbate brain injury in MCAO rats. SGs possess a dynamic structure and exert anti-apoptotic functions in stressed cells. Immediately after an ischemic attack, RBPs translocate from the nucleus to the cytoplasm, triggering SG formation and protecting mRNA from ischemic damage [107]. Reperfusion appears to be crucial for RBPs to return to the nucleus, allowing protein synthesis to resume post-ischemia. Prolonged lack of blood flow recovery leads to sustained translation stagnation, causing severe neuronal damage.

The miRNA m6A methyltransferase methyltransferase-like 3 (*METTL3*)-mediated methylation promotes SG formation in the early stages of acute ischemic stroke. Initially, an MCAO model was established in rats, while oxygen-glucose deprivation/reperfusion (OGD/R) models were developed in primary cortical neurons and PC12 cells [95]. These models revealed increased levels of infarction and apoptosis in the ischemic cortex, accompanied by a significant reduction in SG formation. *Mettl3*-mediated m6A methylation facilitated the maturation of miR-335, which, in turn, enhanced SG formation by degrading the mRNA of eukaryotic translation termination factor (*Erf1*) mRNA and mitigated apoptosis in injured neurons and cells. The RNA-binding protein TDP-43, known to form abnormal cytoplasmic aggregates in the brains of patients with ALS and frontotemporal dementia (FTD), undergoes SUMOylation at lysine position 136 [108]. Studies utilizing a SUMO-mutated TDP-43 K136R protein depicted that SUMOylation modifies TDP-43’s splicing activity, influencing its subcellular localization as well as recruitment to SGs following oxidative stress [108] (Table 1). The rapid assembly of SGs may improve neuronal survival by preserving essential mRNA and/or inhibiting detrimental mRNA once ischemic damage occurs, highlighting the potential therapeutic significance of modulating SG dynamics in ischemic stroke.

Hemorrhagic stroke arises from the rupture of small arteries due to vascular pathologies. Following vessel rupture, hemoglobin infiltrates the brain, inducing pathological processes such as edema, inflammation, and apoptosis, ultimately causing neurological dysfunction [109, 110]. Key contributors to this condition include persistently elevated arterial pressure, which induces cellular-level vascular remodeling, precipitating lipiditis, true arteriolar dissection, aneurysm rupture, and the extravasation of blood under elevated pressure into the deep brain parenchyma. Another significant etiology is cerebral amyloid angiopathy (CAA), marked by progressive accumulation of amyloid protein in cerebral capillaries, arteries, arterioles, veins, and venules, leading to fibrinoid necrosis, weakening of the vessel wall, and subsequent rupture into the cerebral parenchyma. The amyloid buildup in blood vessels is similar to the pathological features of neurodegenerative diseases. Fatal neurodegenerative conditions such as Alzheimer’s disease (AD) [111], ALS [112], coupled with frontal dementia (FTD) [113], are marked by cytoplasmic accumulation of aberrant proteins. RBPs such as TDP-43, FUS, and microtubule-associated protein tau form pathological protein aggregates and indirectly influence SG dynamics through interactions with established SG-related components [114, 115]. These RBPs also participate in cellular stress responses by forming SGs in the cytoplasm, thereby temporarily halting translation to mitigate cellular damage (Table 1).

Stroke and coronary heart disease are related diseases, both caused by atherosclerosis. Before atherosclerosis occurs, there is damage to the inner wall of the blood vessel. On the basis of this damage, lipid deposits are formed, gradually resulting in atherosclerotic plaques, which cause stenosis of the lumen. Therefore, understanding the role of SG in stroke may be important for the treatment of atherosclerosis.

### 5.3 Sepsis

Sepsis, a prevalent post-traumatic complication, manifests as a life-threatening multi-organ syndrome arising from a dysfunctional host response to infection [13]. Patients may succumb to early or late reactivation of immunosuppression or excessive inflammation caused by the primary infection. Studies have highlighted the protective effect of SGs against septic CM dysfunction. Lipopolysaccharide (LPS) activates SGs in CMs through the classical eIF2α phosphorylation pathway. Integrated stress response inhibitor (ISRIB), a broad-spectrum stress response inhibitor, blocks eIF2α phosphorylation, thereby inhibiting SG activation and preventing SG formation, which exacerbates LPS-induced myocardial injury, whereas enhancing SG formation mitigates LPS-induced CM dysfunction [96]. YB-1, secreted by mesangial and immune cells in response to attacks by cytokines, is elevated in the serum of patients with sepsis and tumors [97]. The binding of YB-1 protein to SGs represents a typical cellular response to stimuli such as arsenite, H_2_O_2_, and 45 °C heat shock (Table 1). A large number of studies have confirmed that the increased risk of major cardiovascular events in adult survivors of sepsis is related to typical cardiovascular risk factors, comorbidities, and the characteristics associated with the onset of sepsis. Understanding the protective effect of SGs in sepsis may indirectly provide a new direction for reducing the occurrence of cardiovascular events in the survivors of sepsis.

### 5.4 Atrial Fibrillation (AF)

AF is the most common arrhythmia in clinical practice and serves as an independent risk factor for complications from stroke resulting from cardiovascular and cerebrovascular diseases [116]. The prevailing theory on the pathophysiology of AF is that inflammation and oxidative stress induce atrial electrical and structural remodeling, thereby prolonging and exacerbating the episodes of AF. Research has established a cellular AF model using 600 beats per minute field stimulation. SGs were detected in rapidly paced HL-1 and primary CMs. Overexpression of *G3BP1* in HL-1 CMs significantly reduced ROS levels and calcium overload, while also inhibiting cardiac fibroblast proliferation and collagen synthesis in myocardial fibroblasts [10] (Table 1). These findings emphasize the importance of SGs in cardiac muscle cells and that SGs can significantly inhibit the proliferation of cardiac fibroblasts induced by AngII stimulation. This indicates that the formation of SGs has a protective effect from the complications associated with atrial fibrillation.

### 5.5 Heart Failure (HF)

The highly pathogenic R636S allele of human RNA-binding protein 20 (*RBM20*), which encodes a skeletal muscle-specific nuclear selective splicing factor, was introduced into pigs via genome editing to model dilated cardiomyopathy (DCM) and HF, resulting in abnormal accumulation of RNP granules within muscle tissue. Cytoplasmic *RBM20* RNP granules exhibited fluid-like properties, aligning at regular intervals along cytoskeletal structures, promoting phase allocation of cardiac biomolecules and their fusion with SGs [16]. The normal function of *RBM20* is as a crucial RNA splicing regulatory factor in the myocardium. As an “RNPs assembly regulatory factor”, it inhibits the abnormal aggregation of stress granules (SGs), which form briefly under physiological conditions and quickly disperse after the stress is relieved. In the *RBM20* gene-edited pigs (simulating the human *RBM20* mutant cardiomyopathy), the mutant *RBM20* (such as *RBM20*ΔRRM) loses its normal function: it loses the ability to regulate the assembly of RNPs, resulting in abnormal stable aggregation of SGs (even without obvious external stress, there are still a large number of SGs continuously present in the cardiac muscle cells), and at the same time, other RNPs (such as processing bodies, P-bodies) interact abnormally with SGs, forming a dysfunctional “RNP complex”. In this *RBM20* mutant cardiomyopathy model, SGs play a definite “disease-causing role” rather than a “protective role” (Table 1).

### 5.6 PAH

PAH serves as a severe cardiovascular condition characterized by the abnormal proliferation of smooth muscle cells, leading to excessive collagen synthesis. This results in arterial wall thickening as well as lumen narrowing, which elevates pulmonary vascular resistance and induces pathological remodeling of the pulmonary artery. These changes subsequently cause right ventricular hypertrophy and, in severe cases, death [117]. Studies utilizing the Sugen/hypoxia (SU/Hx) rat model of PAH have revealed increased formation of SG and upregulated expression of SG proteins in the right ventricle and soleus muscle. Treatment with acetazolamide (ACTZ) can restore the contractile phenotype of pulmonary vascular smooth muscle cells, enhance experimental PAH interventions, alleviate disease symptoms, and reduce SG formation in these tissues [17]. Therefore, ACTZ is considered a promising therapeutic target for treating PH by inhibiting SG formation. Research has also highlighted the significant role of various RNAs in the development of PAH (Table 1). The long non-coding RNA LINC00599 is upregulated in the inner layer of the pulmonary artery and regulates SG formation via N6-methyladenosine (m6A) modification, boosting the LLPS of myosin heavy chain 9 (MYH9), thereby promoting proliferation of PASMCs and the progression of PH [18]. Unlike classical mechanisms of long non-coding RNAs, LINC00599 acts as a regulator of m6A-dependent SG dynamics and MYH9-mediated phase separation, offering new insights into the functional roles of long non-coding RNAs in vascular diseases as well as providing a precise therapeutic target for PH [18] (Table 1). In addition, circular RNAs (circRNAs) regulate PASMC pyroptosis through ER stress. Overexpression of circSSR1 inhibits cell pyroptosis *in vitro* and *in vivo* under hypoxic conditions. circSSR1 can promote the translation of its host gene SSR1 through m6A modification, activate ER stress, and induce PASMC pyroptosis [118]. G3BP1 induces the degradation of circSSR1 under hypoxia, suggesting that SGs may contribute to the pathogenesis and progression of PAH under conditions of increased cellular stress (Table 1).

### 5.7 MIR Injury

During acute myocardial ischemia, Sephin1 is a potential key therapeutic target by enhancing the Integrated Stress Response (ISR). It promotes the formation of SGs, increases the number of autophagic vesicles, and inhibits the synthesis of relevant proteins. These actions collectively improve myocardial cell apoptosis and promote autophagy under MIR stress, thereby offering a promising therapy for treating acute myocardial ischemic diseases [98]. ALKBH5 may upregulate SG expression through the cyclic GMP–AMP synthase -stimulator of interferon genes (cGAS-STING) pathway, thereby inhibiting CM apoptosis during diabetic MIR [99]. Therefore, SGs may play a protective role in diabetic MIR (Table 1).

## 6. Targeting SGs to Treat CVD

### 6.1 Targeting Signal Transduction Pathways Formed by SGs

SGs initially form from endoplasmic reticulum-derived structures and regulate key signaling pathways, including those involving eIF4F as well as eIF2α [119]. The mammalian target of rapamycin (mTOR) also participates in these signaling processes. ISRIB, the first identified ISR activator targeting eIF2B, can enhance the activity of eIF2B to simulate the effect of reduced p-eIF2. This blocks the formation of eIF2α phosphorylation-dependent SGs, promotes their rapid disassembly, and restores mRNA translation. ISRIB has demonstrated therapeutic potential across various diseases, such as neurodegenerative diseases, breast cancer, liver cancer, glioma, and leukemia [120]. In CVD, ISRIB rescues reduced calmodulin and SM22α protein levels while decreasing elevated RUNX2 and BMP2 levels in the calcified aorta. It prevents eIF2α phosphorylation and ATF4 elevation, and partially inhibits PERK phosphorylation in the calcified aorta [121]. These findings suggest that eIF2α phosphorylation inhibitors can alter the pathogenesis of vascular calcification by blocking eIF2α/ATF4 signaling, thereby alleviating hypertension and atherosclerosis. ISRIB reduces the incidence of AF by inhibiting the ISR pathway-related cardiac fibrosis, inflammatory macrophage infiltration, and autophagy, while restoring ion channel and TDP-43 expression. Therefore, ISR may have a critical dysfunctional role in the pathogenesis of AF [122].

### 6.2 Targeting PTMs of SGs-Associated Proteins

During the formation of SG proteins, various PTMs, including methylation, ubiquitination, phosphorylation, sumoylation, acetylation, O-GlcNAcylation, and poly (ADP)-ribosylation, critically influence their interactions with SGs and modulate granule assembly, disassembly, fusion, and fission dynamics.

N6-methyl-methyladenosine (m6A), a prevalent RNA methylation modification, plays a critical role in CVD. For example, m6A-mediated miRNA-193a exacerbates sepsis-induced cardiomyopathy, while Mettl3-mediated m6A modification destabilizes coronary plaques [123]. Alternatively, METTL3 regulates miR-335-3p expression to suppress nod-like receptor protein 3 (NLRP3) inflammasome activation, thereby inhibiting microglia activation, blood-brain barrier permeability, and the risk of ischemic stroke [124]. METTL3 also promotes lncRNA-SNHG8 through m6A modification, inducing oxidative stress and myocardial infarction (MI) by regulating ALAS2, and m6A-modified mRNAs are enriched in SGs, enhancing their capacity for phase separation and facilitating larger granule formation [95, 125]. The m6A-binding YTHDF protein promotes SG assembly [126], while METTL3-mediated miR-335 maturation further supports SG formation [95, 123]. This reduces apoptosis in damaged neurons and cells, a potential therapeutic strategy for acute ischemic stroke. Therefore, m6A-modified mRNAs represent promising therapeutic targets for CVD.

Autophagy, ubiquitin-proteasome system (UPS), as well as SUMO-targeted ubiquitin ligase (StUbL), are implicated in SG disassembly [127, 128, 129]. Overexpression of the ubiquitin ligase TRIM21 stimulates K63-linked ubiquitination of G3BP1, inhibiting SG formation, while G3BP1 interacts with autophagy receptors sequestosome 1 (SQSTM1) and calcium-binding and coiled-coil domain-containing protein 2 (CALCOCO2) to promote SG clearance [130]. During heat shock, K63-linked ubiquitination of G3BP1 facilitates its interaction with the ER-associated protein FAF2, which recruits segregase VCP/p97 to extract G3BP1 from SGs, causing their disassembly [131]. The poly-SUM-RNF4 pathway targets RBPs to eliminate misfolded protein aggregates, while the StUbL system may influence SG dynamics by regulating nucleocytoplasmic exchange of SG-associated RBPS. eIF4A2 sumoylation contributes to SG formation, and C9ORF72-ALS-associated proline-arginine dipeptide impairs sumoylation of SG-associated proteins, ameliorating ALS phenotypes *in vivo* [128].

The acetylation of G3BP1 K376 facilitates SG disassembly, a process regulated by HDAC6 and CBP/p300, offering potential therapeutic targets for pathological conditions [132]. Recently, RNA helicase DDX3X was identified as a novel HDAC6 substrate. Under stress, DDX3X undergoes acetylation in its intrinsically disordered region, inhibiting SG formation; conversely, HDAC6-mediated deacetylation of DDX3X promotes SG assembly and maturation [133]. In atherosclerotic *ApoE*^-⁣/-^ mice fed a high-fat diet, G3BP1/2 acetylation impairs SG formation, promoting endothelial marker expression while reducing mesenchymal cell markers [134]. HDAC11 can promote SG formation to facilitate endothelial-mesenchymal transition (EndMT) in atherosclerosis, suggesting SG targeting as a novel therapeutic strategy for this disease [135].

ADP-ribosylation regulates protein function through covalent modification or non-covalent binding of ADP-ribose to substrates [136]. Studies reveal that poly (ADP-ribose) (PAR) is present in SGs, influencing their assembly as well as disassembly. Several PARP family members, including PARP5a, PARP12, PARP14, and PARP15, along with inactive PARP13.1 and PARP13.2, as well as PARGs isoforms PARG99 and PARG102, localize to cytoplasmic SGs [137]. Alphavirus non-structural protein 3 (nsP3) acts as a mono-ADP-ribosylhydrolase, removing PARylation from the SG component G3BP1 to inhibit SG formation [138]. Many proteins in SGs are PARylated or have PAR-binding domains, such as heterogeneous nuclear ribonucleoprotein A1 (hnRNP A1), TAR TDP-43, Ras-GTPase-activating protein SH3 domain-binding protein (G3BP), argonaute family member Ago2, and T-cell intracellular antigen-1 (TIA-1) [139]. Under periods of oxidative stress, Golgi-localized PARP12 translocates to SGs via direct PAR binding [81].

O-GlcNAc modification is crucial for SG formation by aggregating untranslated messenger ribonucleoproteins [140]. During heat shock, the N-terminal region of eIF4GI undergoes dynamic O-GlcNAcylation, trapping stress mRNAs in SGs that persist following stress recovery [141]. Stress-induced O-GlcNAcylation of eIF4GI repels poly(A)-binding protein 1, promoting SG disassembly as well as enabling selective translation of stress mRNAs [142].

Ubiquitination and methylation usually promote SG dissociation and clearance, while SUMOylation, phosphorylation, and acetylation exhibit context-dependent roles in SG dynamics, varying with the involved substrate. These findings underscore the complex interplay of post-translational modifications in regulating SG biology and their therapeutic potential in cardiovascular and neurodegenerative diseases.

### 6.3 Targeting Microtubule-Associated Proteins

Microtubules, key components of the eukaryotic cytoskeleton, are essential for tissue transport and maintaining the stability of CMs. Myocardial ischemia and microtubules damaged by reperfusion injury result in disruption of the balance between polymerization and depolymerization of microtubules in CMs, resulting in loose and broken microtubules, which affect the normal physiological functions of CMs [143, 144].

Recruitment of mRNP in cardiac cytoplasm to fuse into mature SGs requires transport of motor proteins along microtubules, which are involved in cell division, organization of intracellular structures, and intracellular transport. Therefore, microtubule integrity and stability are vital for SG assembly. Microtubule disruption can hinder SG formation. Microtubule stability is mainly regulated by MAPs, including MAP1, MAP2, Tau, and MAP4. MAP1, MAP2, and Tau are primarily neuron-specific. Tau’s excessive phosphorylation and abnormal aggregation are linked to neurodegenerative diseases such as Alzheimer’s disease, FTD, and Parkinson’s syndrome. The SG core protein G3BP2 binds to Tau, preventing its aggregation and thereby reducing the risk of neurodegenerative diseases [145]. SG proteins TIA-1 and TTP bind to phosphorylated Tau and are localized in neurofibrillary tangles in advanced AD and FTDP-17 animal models [146]. MAP4, predominantly expressed in CMs, is often detected in patients with heart disease. After an MI, phosphorylation at the microtubule-binding site of MAP4 causes its displacement from microtubules, leading to cytoplasmic polymer formation. MARK4 regulates CM contractile force by promoting MAP4 phosphorylation and facilitating the entry of angiostatin 2 (VASH2), a tubulin carboxypeptidase, into microtubules for alpha-tubulin dectyrosination [144]. MARK4 knockout mice exhibit significantly improved cardiac function post-MI, suggesting MARK4 may be a promising therapeutic target for improving post-infarction myocardial function. SND1, an SG component protein, colocalizes with microtubules under heat shock conditions. Nocodazole-mediated microtubule disassembly significantly affects the effective recruitment of the SND1 protein to SGs during the stress of heat shock. A complete microtubule cytoskeleton locus is vital for efficient assembly of SND1 granules under heat shock stress, and may facilitate the shuttle of SND1 between foci of cytoplasmic RNA [147].

### 6.4 Small Compounds Targeting SGs

Numerous small-molecule compounds have been identified to influence the process of assembly and disassembly of SGs through different mechanisms. SGs form under specific stress conditions and are tightly regulated by a large number of inflammatory factors, organelle dynamics, post-translational modifications, and microtubule networks. Targeting SG-associated proteins presents a promising path for developing therapeutic strategies against related diseases. Based on the entire dynamic process of SG formation, decomposition, and fusion, we have summarized the compounds that enhance SG assembly (Table 2, Ref. [12, 39, 59, 147, 148, 149, 150, 151, 152, 153, 154, 155, 156, 157]) and those that inhibit SG assembly (Table 3, Ref. [54, 153, 158, 159, 160, 161, 162]), along with their usage, dosage, and mechanisms. These results indicate that the assembly and disassembly of SGs can be pharmacologically regulated at multiple levels. However, most of the current data mainly comes from cultured cells and animal disease models, lacking human tissue samples. Considering that SGs have profound biological effects on different cell types and disease models under various stress conditions, it will be necessary to conduct corresponding clinical translational research in the future.

**Table 2.** **Compounds enhancing SG assembly**.

Category	Compound	Dose	Mechanism	References
Oxidative stress	Sodium arsenite (NaAsO_2_ )	200 µM, 0.5 h	Induce eIF2α phosphorylation	[147]
	Sodium selenite (Na_2_SeO_3_)	1 mM, 1 h	Induce 4EBP1 dephosphorylation	[148]
	Hydrogen peroxide (H_2_O_2_)	200 µM, 20 min	Induce 4EBP1 hypophosphorylation	[149]
	Potassium tellurite (K_2_TeO_3_)	0.6 mM, 3 h	Induce eIF2α phosphorylation	[59]
	Sorbitol	0.4 M sorbitol 150 min	Induce eIF2α phosphorylation	[150]
ER stressor	Thapsigargin	10 μM, 1 h	Endoplasmic reticulum Ca^2+^-ATPase (SERCA) pump inhibitor	[151]
	Doxorubicin	2 µM, 6 h	Induce eIF2α phosphorylation	[152]
	Oxaliplatin	500 µM, 6 h	Induce eIF2α phosphorylation	[152]
	Mitoxantrone	2 µM, 6 h	Induce eIF2α phosphorylation	[152]
	1,4-dithiothreitol (DTT)	1–5 mM DTT, 1 h	Induce eIF2α phosphorylation	[153]
Mitochondrial inhibitor	Malonate	100 mm,1 h	Induce 4EBP1 hypophosphorylation	[154]
	Paraquat	1 mM, 24 h	Induce eIF2α phosphorylation	[12]
	Sodium azide (NaN_3_)	76 mM, 2 h	Induce eIF2α phosphorylation	[155]
	Clotrimazole	20 µM, 0.5 h	Inhibition of the Erk/MAPK pathway	[39]
Effect microtubule	Paclitaxel	0.1 µM, 24 h	Promote microtubule	[156]
	Vinorelbine (VRB)	150 µM, 1 h	Inhibit microtubule formation	[157]
	Vinblastine (VBL)	300 µM, 1 h	Inhibit microtubule formation	[157]
	Vincristine (VCR)	750 µM, 1 h	Inhibit microtubule formation	[157]

**Table 3.** **Compounds inhibiting SG assembly**.

Category	Compound	Dose	Mechanism	References
Destroy microtubule	Nocodazole	2 µg/mL, 2 h	Promote microtubule depolymerization	[158]
Transcriptional inhibitors	Actinomycin D	10 µM, 0.5 h	Unknown	[153]
	Triptolide	30 nM, 0.5 h	Unknown	[153]
Inhibitors of binding to the NTF2L domain of G3BP1/2	G3Ia	50 µM, 1 h	Through antagonism of binding between G3BP1/2 and their binding partners within the NTF2L domain, such as caprin 1 and USP10	[159]
	G3Ib	50 µM, 1 h	Through antagonism of binding between G3BP1/2 and their binding partners within the NTF2L domain	[159]
ATP-competent kinase inhibitor	Staurosporin (RO-31-8220)	10 µM, 1 h	ATP-mimetic inhibitor	[160]
	5′-iodotubercidin (5′-ITU)	10 µM, 1 h	ATP-mimetic inhibitor	[54]
Ribosomal inhibitors	Anisomycin	20 ng/mL, 12 h	Increased cardiomyocyte apoptosis induced by Dox	[161]
Translational protein synthesis inhibitor	Harringtonine	2 µg/mL, 0.5 h	Limit mRNA localization to stress granules	[162]
	Lactimidomycin	50 µM, 0.5 h	Limit mRNA localization to stress granules	[162]
	Rocaglamide A	1 µM, 10 min	Limit mRNA localization to stress granules	[162]
	Puromycin	2 µg/mL, 10 min	Limit mRNA localization to stress granules	[162]
	Emetine	45 µM, 10 min	Limit mRNA localization to stress granules	[162]

It has been confirmed that ISRIB reduces abnormal SG aggregation by blocking the eIF2α phosphorylation pathway, thereby alleviating the inflammatory response and endothelial damage in atherosclerosis. Other summarized SG compounds mainly focus on the organelle dynamics, post-translational modifications, and microtubule networks of SGs throughout their entire life cycle, or are derived from other diseases. Currently, they are less frequently used in CVD research. Therefore, this could serve as a stimulus for further investigations into the role of SGs in CVD.

### 6.5 The Inflammatory Response is the Basis for Both the Occurrence of CVD and the Formation of SGs

#### 6.5.1 Inflammation and CVD

Inflammation serves as a significant risk factor for CVD, and controlling its risk factors is crucial for reducing the incidence of cardiovascular events. RBPs can bind to AU-rich elements (ARE) in the 3^′^UTR of mammalian mRNAs, thereby regulating their transcription. Many inflammatory cytokine mRNAs contain conserved or semi-conserved AREs in their 3^′^UTR, offering a potential target for reducing inflammation [163]. Myeloperoxidase (MPO), a marker of oxidative stress as well as inflammation, is located in the basophilic granules of neutrophils and monocytes. It promotes chronic inflammation and local tissue damage through the generation of reactive oxygen species. Studies have demonstrated that MPO can destabilize atherosclerotic plaques and modify low-density and high-density lipoproteins that contribute to the progression of atherosclerosis and ischemic heart disease [164]. Rheumatoid arthritis (RA) and atherosclerotic CVD have been shown to promote interleukin (IL)-1 and IL-6 inflammatory pathways, activate tumor necrosis factor (TNF) pathways, Janus kinase-signal transducer and activator of transcription (JAK-STAT) signaling pathways, and inhibit IL-1 and IL-6 to reduce the risk of CVD [165]. Following tissue injury, activation of the NLRP3 inflammatorome leads to the release of cytokines interleukin-1β and IL-18, finally causing inflammatory cell death, specifically pyroptosis. NLRP3 inflammasome plays a pivotal role in the pathogenesis of various CVDs, including hypertension [166], MI, CM, and HF. Exploiting NLRP3 inhibitors represents a promising approach to mitigate CVD. Targeting the NLRP3 inflammasome with colchicine, an NLRP3 inhibitor, has recently been shown to significantly reduce cardiovascular events in patients with chronic coronary artery disease.

#### 6.5.2 Inflammation and SGs 

In mucosal inflammation, pro-inflammatory cytokines such as IFN-γ, as well as TNF-α, trigger phosphorylation of eIF2α, which induces SG formation. Within these SGs, HSP70 mRNA is encapsulated, leading to a reduction in HSP70 expression [57]. When macrophages are subjected to stressors that can induce SGs and the NLRP3 inflammasome, including cytoplasmic Poly (I:C) stimulation, they preferentially form the NLRP3 inflammasome. This is achieved by sequestering G3BP into the inflammasome and by increasing intracellular K+ levels, thereby avoiding SG assembly [167]. The SG protein DDX3X interacts with NLRP3 to promote inflammasome activation. Both NLRP3 inflammasome-dependent pyroptosis and pro-survival stress granules require the involvement of DDX3X [168]. DDX3X is located at the “surviving-pyrodeath” intersection and becomes a checkpoint molecule for determining the fate of cells, which can be engineered as a drug target. HF is associated with an enhanced inflammatory response, and NLRP3 inflammasome activation is a vital mechanism that links mitochondrial function and integrity to innate immunity. During HF’s progression, increased mitochondrial damage linked to higher oxidative stress, as well as impaired quality control via autophagy/mitochondrial autophagy, can lead to myocarditis. mtDNA and ROS have been identified as triggers of the inflammatory response. The regulation of innate immunity by mitochondrial function is being increasingly recognized in cardiac as well as non-cardiac diseases [50, 51, 169].

### 6.6 Limitations

Despite the comprehensive overview of stress granules (SGs) in cardiovascular physiology and pathology provided in this review, several critical limitations should be noted.

First, most mechanistic insights are obtained from *in vitro* models and experimental animals, while direct evidence from human cardiovascular tissues is scarce. Species differences and cellular microenvironmental heterogeneity may limit the translational potential of these findings to clinical practice.

Second, the dual role of SGs (protective versus pathogenic) in different CVDs and different pathological stages remains ambiguous. The molecular determinants governing the transition from reversible adaptive SGs to persistent pathological aggregates in the heart and blood vessels require further clarification.

Third, current studies focus primarily on G3BP1/2-mediated SG assembly, whereas the functions of other SG components, non-coding RNAs, and post-translational modifications in CVDs are largely underexplored. The compositional heterogeneity and cell-type specificity of SGs in cardiomyocytes, endothelial cells, vascular smooth muscle cells, and macrophages have not been systematically characterized.

Fourth, SG-targeted therapeutic strategies are still at the preclinical stage. Most small-molecule modulators lack cardiac specificity and may cause off-target effects. Long-term safety, efficacy, and optimal administration regimens in chronic CVD models remain to be verified.

Fifth, the crosstalk between SGs and other critical pathways—such as autophagy, inflammation, mitochondrial homeostasis, and epigenetic regulation—remains incompletely understood. Integrated regulatory networks rather than single pathways need to be further investigated.

Finally, there is a lack of clinical biomarkers and *in vivo* detection methods for monitoring SG dynamics in CVD patients, which hinders patient stratification, prognosis evaluation, and translational drug development.

## 7. Conclusions

SGs, a type of membrane-less organelle, are dynamically formed by cells in response to stress stimuli. This stress response system is transient and typically reversible upon removal of the stressor. However, prolonged or chronic stress can lead to the accumulation of persistent SGs, which may contribute to pathological changes and the onset of other related diseases. Certain disease-related proteins may participate in the formation or clearance of SGs under specific conditions, further complicating their role in the pathology of various diseases. The pathophysiology of CVD may arise from the inherent vulnerability of cellular metabolism, in which RBPs regulate RNA metabolism by condensing into membrane-less organelles, predisposing them to pathological aggregation.

In this article, we delve into the diverse physiological activities and molecular mechanisms of SGs in CVD. The molecular mechanisms underlying the fusion and fission of SGs during pathological processes remain an intriguing and promising area of research. Exploring the potential association between the heterogeneity of atherosclerotic plaques, as well as phenotypic heterogeneity in CVD, is another promising field. Revealing the impact of SG dysfunction on the functions of other organelles via organelle interactions represents an exciting and rapidly evolving research field. Given the inherent diversity of the dynamic nature of membrane-less organelles like SGs, elucidating their internal structure poses a significant challenge; however, utilizing high-resolution imaging techniques can help to explore these highly dynamic biological systems. These techniques enable the capture of dynamic changes in membrane-less organelles and the interpretation of assembly patterns of proteins and RNA molecules within them, providing valuable insights into their function and regulation in health as well as disease.

This article systematically reviews the biological characteristics, cellular interaction networks, and pathological physiological functions of SGs in CVD. The core advantages are significant:

Multi-dimensional integration framework: For the first time, a three-dimensional system of “SGs’ life cycle—cellular interaction—CVD pathological mechanism” was constructed, clarifying the dynamic regulatory relationships between SGs and the endoplasmic reticulum, mitochondria, Golgi apparatus, and lysosomes, filling the gap in the review of SGs in the field of multicellular organ coordination in CVD.

Comprehensive disease coverage and responsible mechanisms: Covering 7 core CVD types such as atherosclerosis, stroke, and PAH, through the logical chain of “molecular targets—signaling pathways—disease phenotypes”, the dual roles (protective function/pathological accumulation) of SGs were analyzed, particularly highlighting key regulatory mechanisms such as LLPS and PTMs.

Systematic treatment strategies: From four dimensions of signaling pathways, PTMs modifications, microtubule-related proteins, and small molecule compounds, a targeted treatment system for SGs was constructed. The compound action mechanisms and doses were presented in a tabular form, with transformation references, helping to enhance their clinical value.
